# The Hemodynamic Parameters Values Prediction on the Non-Invasive Hydrocuff Technology Basis with a Neural Network Applying

**DOI:** 10.3390/s22114229

**Published:** 2022-06-01

**Authors:** Marina Markuleva, Mikhail Gerashchenko, Sergey Gerashchenko, Robert Khizbullin, Igor Ivshin

**Affiliations:** 1 Medical Cybernetics and Computer Science Department, Penza State University, 440026 Penza, Russia; marina--mark@mail.ru (M.M.); cts.com@yandex.ru (M.G.); mpo@list.ru (S.G.); 2 Kazan State Power Engineering University, Krasnoselskaya, 51, 420066 Kazan, Russia; ivshini@mail.ru

**Keywords:** blood pressure, pressure, pulse wave, hemodynamic parameters, hydrocuff technology, feature selection algorithm, multiparameter feature space, machine learning

## Abstract

The task to develop a mechanism for predicting the hemodynamic parameters values based on non-invasive hydrocuff technology of a pulse wave signal fixation is described in this study. The advantages and disadvantages of existing methods of recording the ripple curve are noted in the published materials. This study proposes a new hydrocuff method for hemodynamic parameters and blood pressure values measuring. A block diagram of the device being developed is presented. Algorithms for processing the pulse wave contour are presented. A neural network applying necessity for the multiparametric feature space formation is substantiated. The pulse wave contours obtained using hydrocuff technology of oscillation formation for various age groups are presented. According to preliminary estimates, by the moment of the dicrotic surge formation, it is possible to judge the ratio of the heart and blood vessels work, which makes it possible to form an expanded feature space of significant parameters based on neural network classifiers. This study presents the characteristics accounted for creating a database for training a neural network.

## 1. Introduction

The development and implementation of information technologies into medical practice has made it possible to expand the diagnostic prospects for hemodynamic parameters analysis for the purpose of cardiovascular system pathological conditions early diagnosis and adequate therapy conducting. Currently, it is possible to measure more than twenty indicators characterizing the blood through the vessels movement and the cardiovascular system general state.

The researchers refer to the primary hemodynamic parameters as heart rate (HR) and blood pressure (BP), while the extended hemodynamic parameters include stroke volume (SV), cardiac output (CO), total peripheral resistance (TPR), and a number of others [1].

The neural networks implementation for diseases diagnosis is an interesting and promising direction. Recent achievements in the field of laboratory diagnostic equipment and the use of computer modeling methods open new possibilities for cardiovascular system (CVS) monitoring. Interest in this topic is growing every year. Research in this field published in scientometric citation databases: Pubmed, Scopus, and Web of Science focus on such aspects as: mathematical modeling of human CVS activity [2,3,4,5], biomedical image processing [6,7], medical decision support systems [8,9,10], and diseases diagnosis [11,12,13]. The main idea of this kind of research is to show the relationship of blood flow hemodynamic parameters with the vascular wall stiffness, which in turn, characterizes the CVS functional state and allows to identify pathological changes in the vessels.

The problem of hemodynamic parameters monitoring in emergency medicine was successfully solved with the use of resuscitation bedside monitors [14]. If it is necessary to confirm the primary diagnosis, ultrasound examination [15] or magnetic resonance imaging [16] is indicated. The problem of early diagnosis and systematic monitoring of therapy dynamics for cardiovascular system diseases remains unresolved.

Researchers are attempting to solve the task of blood pressure parameters daily monitoring and hemodynamic values by using oscillator-type devices using an air occlusive cuff and photoplethysmography (PPG) [17,18]. Recently, these methods have been severely criticized by researchers, doctors, and engineers, so in [19] the disadvantages of these methods are presented in detail. Despite the low accuracy, the PPG method has not lost popularity, this is due to the simplicity of implementation and the lack of alternative measurement methods for wearable devices.

The artificial neural networks and machine learning technologies implementation for the diseases diagnosis is an interesting and prospective direction [20,21,22,23,24]. Deep learning artificial neural networks have recommended themselves well in many diagnostic medicine fields. Research in the field of predicting blood pressure parameters by applying deep recurrent neural networks is a new direction in cardiovascular system diagnosis. The studies published by Su et al., 2018 [25], which raised the problem of the need for frequent calibration of the model for estimating blood pressure based on PPG in order to maintain accuracy at a sufficient level for a long time, should be noted. A synchronously recorded PPG and electrocardiosignal were used as input data for training the neural network, which in turn complicates the process of data collection and its subsequent processing.

In the paper by Choudhury et al., 2020 [26], an extensive review of world research in the field of PPG collection and processing with machine learning applying was made. In this study, a new approach in recording the contour of the pressure pulse wave is proposed. This study raises the question of the need to form a multiparametric feature space for training a neural network in order to increase the accuracy of prognostic data on the presence and dynamics of the cardiovascular system pathologies development.

The article structure is presented as follows: Section 2 introduces materials and methods, where the authors emphasize factors influencing the multiparametric feature space for the operation of a neural network for the purpose of predicting hemodynamic parameters. Section 3 presents a block diagram of the «Hemodin1» hydrocuff device, as well as the results of field experiments, with a preliminary review. Section 4 discusses the results and possibilities of forming a multiparametric feature space for the functioning of a neural network in order to predict hemodynamic parameters, Section 5 completes the work and reflects the prospects for further research in the field of hydrocuff technology application for measuring blood pressure and hemodynamic parameters.

## 2. Materials and Methods

The process of assessing systolic blood pressure (SBP) and diastolic blood pressure (DBP) with various types of devices is associated with a significant influence of factors that reduce the reliability of the values obtained, these primarily include anatomical features.

One of the reasons for the need to modernize existing methods for assessing hemodynamic parameters and determining blood pressure values is the significant errors in the data obtained.

The existing algorithm underlying the pulsation curve processing is estimated for the average statistical data of patients. It is known that the process of transferring pressure from the artery to the cuff depends on the anatomical features of the forearm or wrist structure.

Another reason is related to the influence of breathing on the pulse curve contour as a consequence of a change in the pulse wave signal amplitude (PW) [27,28].

The third reason is related to the need to form a multiparametric feature space for determining critical conditions and assessing the dynamics of the pathological processes development in the cardiovascular system (CVS) based on neural network technologies.

It is not possible to estimate the values of corrections in order to eliminate their influence by traditional filtering methods [29,30] and removing trends since such transformations cause distortion of the pulse wave contour and amplitude-frequency characteristics underlying the main hemodynamic parameters calculation.

In this regard, a technique based on neural network applying is proposed, which allows, on the basis of training, to produce the effective adjustments to the blood pressure values and other hemodynamics parameters. The neural network is based on learning on a multiparametric feature space that characterizes the CVS state.

Such parameters can be weight-dimensional anatomical parameters such as: age, gender, height, weight, waist circumference, arm circumference, and body mass index.

Another group of indicators forming a multiparametric feature space are hemodynamic parameters: cardiac output, stroke volume, pulse wave propagation velocity, stiffness index and many others. Currently, it is possible to present up to thirty indicators characterizing the CVS state [31,32]. When calculating vital signs of hemodynamics, the values of SBP and DBP are used in the algorithms of their calculation. Inaccuracies and errors in their assessment entail inaccuracies in the hemodynamic parameters values and lead to a distortion of the picture of the disease.

Currently, oscillatory methods [33,34,35,36] do not allow obtaining accurate values of SBP and DBP due to the small amplitude of oscillations. An improved method is the use of hydrocuff technology for the pulse wave contour formation and recording. At the Department of “Medical Cybernetics and Informatics” of Penza State University, research is being conducted on hydrocuff technology [37] for fixing the pulse wave contour to further analyze the hemodynamics and blood pressure parameters.

The disadvantage of the air compression cuff, widely used in oscillator-type tonometers, is the small amplitude of the generated oscillations (1–3 mm Hg). Against the noise background, it is quite difficult to establish peak values of pulse waves underlying the algorithms for obtaining hemodynamic parameters. The use of filtering procedures, trend removal, and smoothing of the envelope of oscillation peaks lead to significant errors in determining pressure values for characteristic points.

The authors of this study upgraded the existing method by applying two chambers of the same type into which liquid is pumped. The oscillations processing is performed on the sensor’s differential activation basis. This makes it possible to implement a direct method of fixing systolic and diastolic pressure levels with a significant reduction in the effect on noise readings, which allows to obtain a more reliable pulse wave contour.

The technical result is achieved due to the fact that an incompressible medium, a liquid, is selected as the working medium, which increases the reliability of the qualitative representation of the pulse wave contour and, as a result, the determination of hemodynamic parameters and blood pressure. A more detailed description of hydrocuff technology is undesirable due to the know-how disclosure.

**Figure 1:**
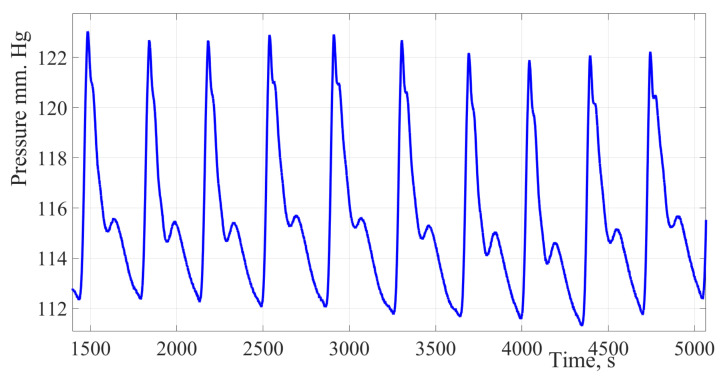
Pulse wave signal recorded using hydrocuff technology. The presented fragment shows the presence of a respiratory component, which leads to a change in the oscillation’s amplitude. When finding an oscillation with the maximum amplitude, it is necessary to take into account the influence of the respiratory component.

The use of a hydrocuff allows to increase the oscillations amplitude up to 20 mm Hg. This creates prerequisites for improving the accuracy of the SBP and DBP assessment. Figure 1 shows a fragment of the blood pressure pulsation signal of a thirty-year-old female patient, the cuff was installed on the forearm.

The study included 30 potentially healthy people (15 women and 15 men): average age, 45 ± 2 years; average height, 175.5 ± 5.7 cm; average weight, 80.3 ± 4.2 kg; average heart rate, 80 ± 8 beats/min; average systolic blood pressure, 119 ± 4 mmHg; and the average diastolic blood pressure, 82 ± 4 mmHg. All participants were informed about the study and provided informed consent. The study was developed in accordance with the Helsinki Declaration and approved by the local ethics committee. All measurements were performed during working hours while observing usual lifestyle. The study involved people who did not take any medications.

Figure 1 shows a fragment of the blood pressure pulsation signal of a thirty-year-old female patient. The cuff was installed on the forearm, the signal obtained by the authors of the study using a new hydrocuff technology for the oscillation’s formation. The signal is characterized by a significant oscillation’s amplitude ranging from 20 to 25 mm Hg, which is almost 50% of the pulse pressure value. The signal is also characterized by a low level of high-frequency interference and a high degree of the pulse wave contour reproducibility.

Due to hydrocuff technology oscillation’s formation, these changes in amplitudes have a significant impact on the estimating values of hemodynamic parameters. In this regard, it is necessary to take into account dynamic changes in amplitudes to stabilize the pulsations amplitudes of the pulse wave contour.

The third group involved in the formation of a multiparametric feature space includes processes that characterize the formation of pathological changes in hemodynamic parameters, allowing to observe their dynamics and compare the values of the forecast with the values recorded in the early stages. As a result of measurements, an array of data was generated for training and testing a neural network.

Taking into account these factors allow to create conditions for the development of systems for assessing the hemodynamics and blood pressure parameters with increased accuracy.

**Figure 2:**
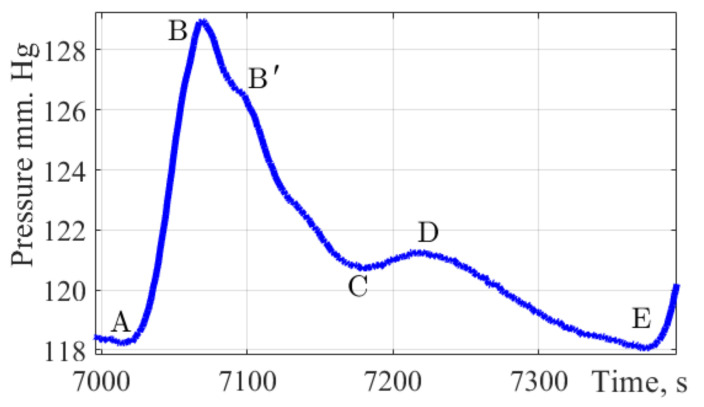
The pulse wave signal fragment. The figure shows a fragment of a single pulse wave contour obtained by the authors of the study, with characteristic points of dynamic changes in pulse pressure applied A, B, B′, C, D, E.

Figure 2 shows a single pulse wave with marked characteristic points A, B, B′, C, D, and E.

This form combines well with the form of the pulse wave in the brachial artery. It allows to present time intervals for characteristic points A, B, B′, C, D, and E which are important parameters in assessing the state of the cardiovascular system and the functions of the left ventricle.

Point A characterizes the cardiac cycle beginning. Peak B is the main amplitude of the pulse signal caused by the cardiac discharge of blood from the ventricles during systole. The rate of wave rise characterizes cardiac output, arterial resistance, and the vascular wall elasticity. Peak B′ is often not visible on pulse wave signals; according to a group of authors [38] this peak reflects the moment when the blood ejection by the left ventricle stops. Its position is influenced by the values of peripheral vascular resistance, the elasticity of the vascular bed. Peak C reflects the moment of systole termination and the beginning of the diastolic phase. The position of this peak is influenced by peripheral vascular resistance. Peak D characterizes the moment when the valve closes and the pressure increases due to the elastic properties of the arteries. The peak E is the end moment of the diastolic phase and the indicator of the final diastolic intravascular pressure.

For the early diagnosis of diseases such as hypertension, atherosclerosis, and other vascular pathologies, it is relevant to determine the following amplitude-time parameters of the pulse wave contour.

The pulse wave amplitude, has no normative values, is estimated in dynamics, and measured in relative units.

The amplitude of the dicrotic wave. Normally, it is ½ of the pulse wave amplitude value, also measured in relative units.

The incision height, normally 2/3 of the pulse wave amplitude value, is measured in relative units.

The dicrotic wave index is measured as a percentage; the normative value is 63–73%.

The duration of the pulse wave anacrotic phase; measurement unit: seconds.

The duration of the pulse wave dicrotic phase; measurement unit: seconds

The duration of the exile phase. A parameter reflecting the diastolic activity of blood vessels; measurement unit: seconds.

The duration of the exile phase. A parameter reflecting the diastolic activity of blood vessels; measurement unit: seconds.

Pulse wave duration; measurement unit: seconds.

The rising wave index. This reflects the filling phase in the systolic period of the cardiac cycle. The standard value corresponds to 15–24%.

Filling time; measurement unit: seconds. The standard value is in the range of 0.06–0.12 s.

The cardiac cycle systole phase duration; measurement unit: seconds.

The cardiac cycle diastole phase duration; measurement unit: seconds.

The cardiac cycle diastole phase duration; measurement unit: seconds.

Pulse wave reflection time; measurement unit: seconds. The standard value is in the range of 0.03–0.04 s.

Heart rate; measurement unit: beats per minute.

Despite the diagnostic significance of the pulse wave contour with its characteristic points, many normative values of hemodynamic parameters have yet to be determined. The absence of these values is due to the complexity of forming a database of pulse wave contours in normal and pathological conditions. 

The above-mentioned components of pulse wave signals can be used to evaluate the characteristic points of the pulse wave contour. In this regard, it is important to focus on the study of the pulse wave contour and its characteristics, which make it possible to form a multiparametric feature space for machine learning of neural network classifiers.

## 3. Results

Pulse wave signals were received using the «Hemodyn1» device. The pressure signal processing algorithm was implemented as a virtual instrument using LabVIEW software. Graphic processing was performed using the MATLAB R2017b software as part of the study, taking into account the age and health status of the participants.

**Figure 3:**
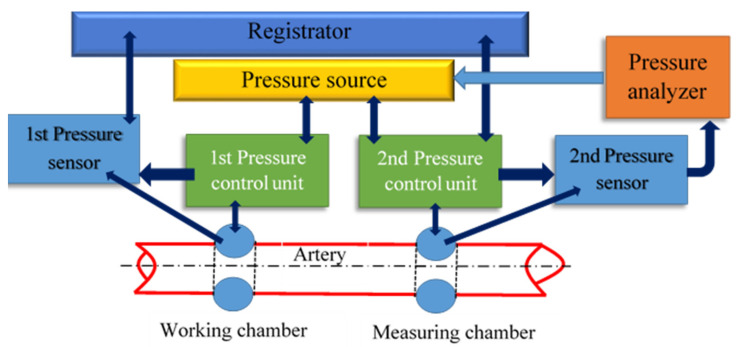
The block diagram of the «Hemodyn 1» device, for assessing the cardiovascular system state. It consists of a two-chamber compression hydraulic cuff, a pressure source (Pressure source), two pressure sensors (Pressure sensor), two pressure control units (Pressure control unit), a pressure analyzer (Pressure analyzer), and a registrar (Registrator).

To study the process of a multiparametric feature space formation and to prove the possibility of predicting the hemodynamic parameters values based on non-invasive hydrocuff technology using a neural network, the authors used the hydrocuff device «Hemodin1», the block diagram of which is shown in Figure 3.

The pressure source contains a reservoir with a given volume of liquid and a device for supplying liquid to the hydrocuff chambers. A two-chamber compression hydrocuff is installed on the patient’s forearm before starting the measurement.

When creating pressure, the liquid from the pressure source enters the working chamber through the first control unit. Excessive pressure is created in it and oscillations are formed caused by the passage of pulse waves under the cuff [37].

The figures below show the pulse wave contours of patients from different age groups registered by the «Hemodin 1» hydrocuff device.

**Figure 4:**
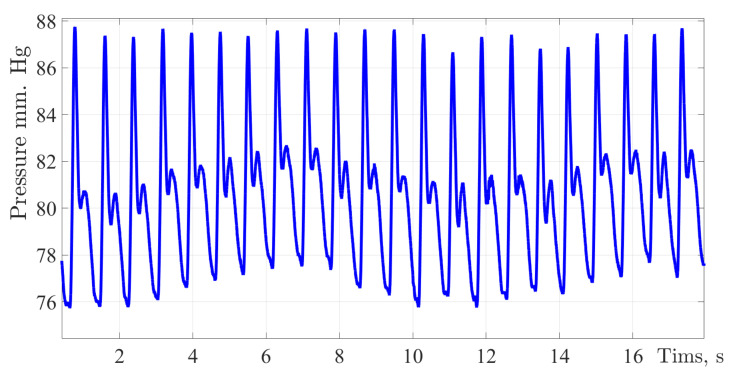
A typical fragment of a pulse wave signal recording obtained during the experiment process. Along the ordinate axis, the values of blood pressure in mmHg are deposited, and along the abscissa axis is the time with a sampling frequency of 512 Hz.

**Figure 5:**
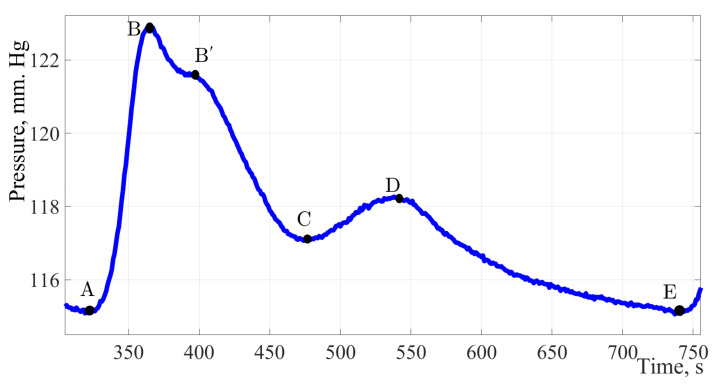
The pulse wave signal fragment of a 20-year-old young men without identified pathologies. Considering the fragment of the pulse wave of a twenty-year-old patient, it can be noted, in addition to the classical peaks, a characteristic sub-lash is marked on contour B′ on the segment of the curve from point B to point C.

**Figure 6:**
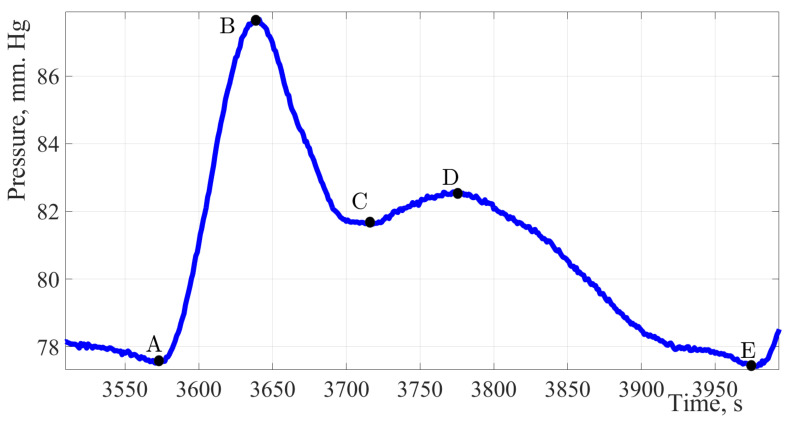
The pulse wave signal of a woman 30 years of age.

**Figure 7:**
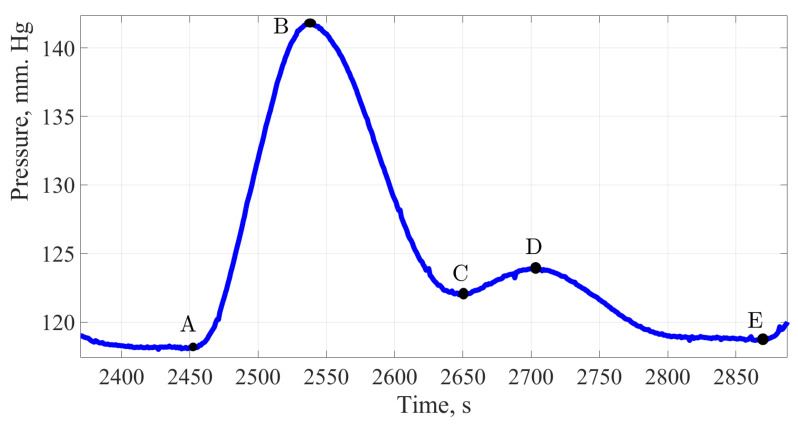
The pulse wave signal of a man 60 years of age.

Figure 4, Figure 5, Figure 6 and Figure 7 were obtained by the authors of this study as part of the development of a system for predicting the hemodynamic parameters values based on non-invasive hydrocuff technology with a neural network applying.

Figure 5 shows the pulse wave signal of a patient with no pathologies identified and no undergoing treatment by a cardiologist, in other words, potentially healthy.

The factors of formation and diagnostic significance of this peak are to be determined during an extended study of a group of patients 18 to 21 years of age.

The maximum amplitude of the presented signal is 8 mm Hg. The amplitude of the dicrotic burst is 3.5 mm Hg, which is 44% of the total amplitude of the signal and indicates a high elasticity of the vessel wall. Having estimated the duration of one cardiac cycle, it can be concluded that 64% of the cardiac cycle time falls on the work of the vessels and only 36% on the work of the heart muscle.

Figure 6 shows the pulse wave signal of a patient without identified pathologies and complaints of violations in the CVS work.

Analyzing the treated contour of the pulse wave, it can be stated the high amplitude of the signal, about 12 mmHg, which characterizes such a hemodynamic parameter as cardiac output, it can also be noted a good response of the artery walls to extensibility under the influence of shock volume, as evidenced by a dicrotic surge on a single pulse wave.

The amplitude of the dicrotic surge was 41.6% of the total amplitude, there is a decrease in vascular response compared with indicators of a younger age. More detailed calculations and in-depth analysis may offset these discrepancies. The working time of the vessels relative to the total duration of the cardiac cycle was almost the same and amounted to 64%, while the work of the heart accounted for 36%.

Figure 7 shows the pulse wave signal of a men aged 60 years. At the time of the study, the man was not taking any medications that affect blood pressure indicators.

Analyzing the contour of the pulse wave of an age-related patient in the same way as in previous cases, the shape of the pulse wave resembles the classical one, there are two waves and all the main characteristic points, a distinctive feature can be called a high amplitude of 22 mmHg; however, the amplitude of the dicrotic surge was 5 mm Hg, this is only 22.7%. This indicates the process of reducing the vascular bed elasticity. The main work is performed by the heart muscle, the weak work of the vessels provides an additional load on the left ventricle of the heart. The same fact confirms the duration of the cardiac cycle, the share of vascular work accounts for 49.4% of the time.

The presented results of recording the single pulse waves prove the individuality of the pulse wave contour and the fact that age-related changes in the elasticity of the vascular wall affect the work of the entire CVS.

**Figure 8:**
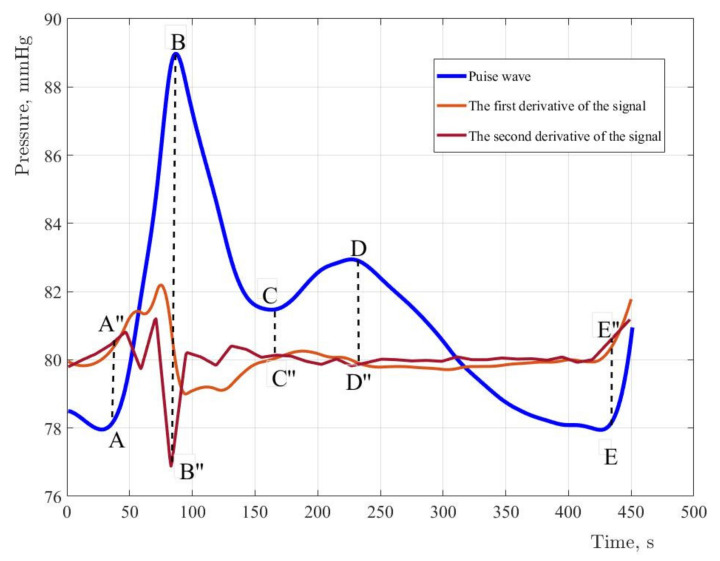
The original signal and derivatives of the first and second order with normalized values and coefficients. For the first derivative, the coefficient is assumed to be equal to five. For the second derivative, the coefficient is fifty. The coefficient determines the steepness of the amplitudes of the derivatives.

In Figure 2, the characteristic elements of the pulse wave are marked with symbols: anacrotic rise (A–B), and dicrotic period (B–E). The pulse wave character depends on the systolic output, the blood flow intensity, blood viscosity, and the vascular wall’s state. Due to the high diagnostic information content of the pulse wave contour, it is necessary to implement special methods which allow to reduce the error of fixing characteristic points on the pulse wave contour. The study used a spline interpolation technique based on changing the position of the characteristic points of the selected fragment of the curve. Signal processing was performed in MATLAB R2017b. In order to determine the characteristic points of the pulse wave contour, the pressure curve must be analyzed by the first and second derivatives of the signal [8]. Figure 8 shows the results of determining the first and second derivatives of the pulse wave signal.

**Figure 9:**
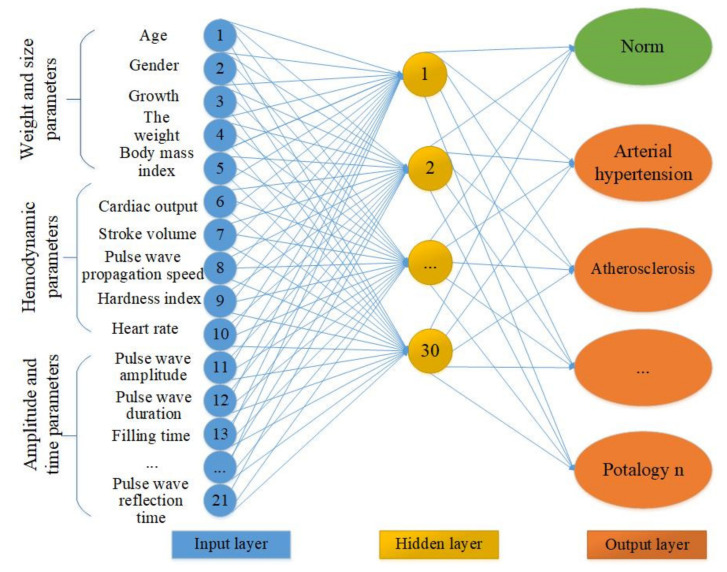
The neural network architecture variant. The under development neural network contains three groups of data in 21 input neurons. The hidden layer and the output layer with pathological conditions.

The use of neural networks when making a diagnosis reduces the time of research and simplifies the task for medical personnel. To form a multiparametric feature space, the authors of this study proposed the structure of an artificial neural network. Figure 9 reflects the neural network architecture variant. The input layer is represented by the three groups of parameters above: mass-dimensional, anatomical, hemodynamic, and amplitude time. 

Determining the sufficient number of neurons in the hidden layers is an important task. A small number of neurons complicates the learning procedure. A large number of neurons leads to an increase in training time and the effectiveness of its tuning. To find the rational number of neurons of the hidden layer of a neural network with the structure of a multilayer perceptron, it is necessary to study the indicators of sensitivity and specificity of all network outputs, which the authors of this study have yet to implement. It is possible that the number of neurons in the hidden layer will decrease as a result of further research. The output layer of the proposed neural network contains information about the normal state and pathologies of the cardiovascular system.

**Figure 10:**
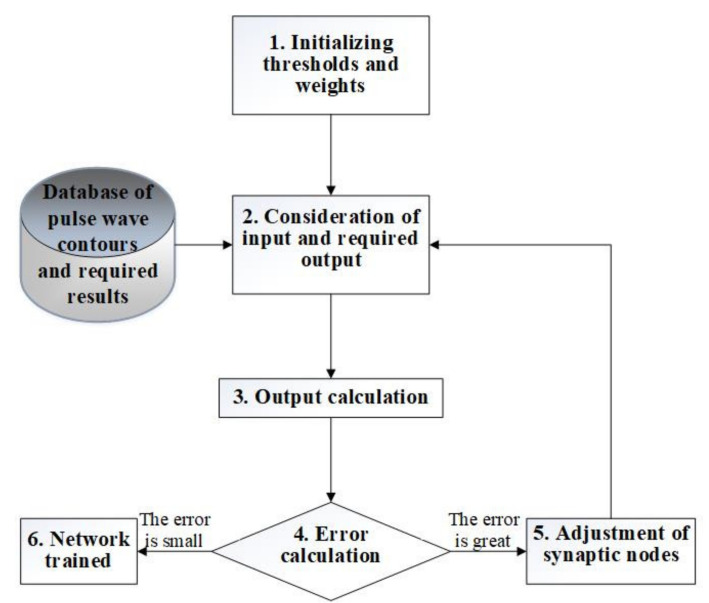
A variant of the neural network learning algorithm.

To train an artificial neural network, it is possible to use an error back propagation algorithm [39]. The variant of the neural network learning algorithm proposed in Figure 10 involves calculating the error of both the output layer and each neuron of the learning network and the correction of the weights of neurons in accordance with their current values.

## 4. Discussion

In this study, the authors recorded the pulse wave contours of citizens of different age groups and analyzed the characteristic points position of the pulse wave contour. In the study, it was aspired to evaluate the factors influencing the SBP and DBP determination accuracy, as well as to evaluate the possibility of forming a multiparametric feature space based on preliminary collection of information about the patient anatomical features and the pulse wave contour obtained by hydrocuff technology applying [37]. The recorded contours of pulse waves of citizens from different age groups clearly demonstrated changes in the pulse wave contour with increasing age of the patient, which indirectly confirmed an increase in the stiffness of the vascular wall. Moreover, in the course of the conducted research, it was desired to substantiate an increase in the quality of the received pulse waves contour. The graphs presented in the paper reflect the original signals that did not pass the filtering stage. For the study results, a large number of pulse wave signals were collected, which are of scientific interest in terms of cardiovascular pathologies diagnostics. The beginning to the formation of BP oscillograms recorded by a device implementing the hydrocuff technology, which was performed for the first time, is set. The obtained unique recordings of arterial pressure pulse waves, open certain prospects in terms of understanding the process of the heart and vascular bed performing.

During the study, the amplitude and time characteristics of the registered signals were evaluated. The obtained preliminary data showed an increase in amplitude with an increase in age, this fact needs to be checked on a larger sample. Moreover, in the conducted research’s result, the task of assessing the effect of pathological changes in the cardiovascular system on the time of a complete cardiac cycle arose. The considered issue of the respiratory organs work influence on the pulse waves contour requires an extended study with the involvement of additional equipment a chest respiratory sensor. The conducted research only confirmed the respiratory system’s effect on the pulse wave contour in all age groups. Studies on the selection of the respiratory component of the signal were not conducted. Hartmann V et al., 2019 [40], published results concerning the determination of the installation location of a photoplethysmographic sensor to accurately calculate the respiratory rate. The Hartmann V et al. study results provide additional prospects for hydrocuff technology application in the field of diagnostics of the respiratory system work and possible pathological conditions identification.

In this study, the authors did not set the task of comparing the developed hydrocuff technology with well-known published works on the assessment of blood pressure and hemodynamic parameters using PPG and ECG signals. The authors propose to use hydrocuff technology to form a high-quality pulse wave contour. 

## 5. Conclusions

This study substantiates the need for the formation of a multiparametric feature space for the purpose of early diagnosis of CVS pathologies. Groups of features are provided that allow forming a large data array for training a neural network. A variant of a multilayer neural network is proposed to increase the prognostic accuracy of the intended diagnosis. The complexity in the formation of a multiparametric feature space is associated with the need to create an extensive database of pulse wave contours characterizing various types of pathologies. The use of hydrocuff technology leads to a complication of the device design compared with PPG, due to the use of expensive microprocessors used to solve neural network classification tasks.

As a result of tests, the contours of the pulse waves were obtained and characteristic points were marked. In this study, a reliable, accurate but simple mechanism is proposed that allows to extract clinically significant signs from the pulse wave curve by taking into account the position of characteristic points on the curves.

The conducted research results indicated a high sensitivity of perception of pressure changes in the artery under the cuff. This has advantages associated with an increase in the pulsations amplitude of small distortions of the pulse waves contour. High sensitivity makes it possible to capture the influence of the respiratory component on the amplitude characteristics of pulse waves. In this regard, the task of eliminating the modulations of the respiration amplitudes arises. For practical use of the proposed technology, it is necessary to develop an algorithm for extracting respiratory amplitudes from the pulse wave contour. This will allow to isolate and take into account the respiratory component in order to exclude the respiratory system work influence on hemodynamic parameters. It will expand the functionality of hydrocuff devices in the field of diagnostics of the respiratory system for determining the frequency and depth of breathing, which is an urgent problem in pulmonology.

## Data Availability

The source data of the pulse wave signals used in the study is in the public domain. Available on: GitHub, https://github.com/marinamarkuleva/The-Hemodynamic-Parameters-Values-Prediction-on-the-Non-Invasive-Hydrocuff-Technology-Basis-with-a-.git (accessed on 20 May 2022).
